# Multimodal brain MRI and clinical data in olfactory groove meningioma: a prospective data report

**DOI:** 10.3389/fradi.2026.1809871

**Published:** 2026-05-08

**Authors:** Elena Filimonova, Anton Pashkov, Galina Moysak, Azniv Martirosyan, Vladimir Kurilov, Aleksandra Poptsova, Renata Morozova, Jamil Rzaev

**Affiliations:** 1 Federal Neurosurgical Center, Novosibirsk, Russia; 2 Novosibirsk State Medical University, Novosibirsk, Russia; 3 Novosibirsk State University, Novosibirsk, Russia; 4 Novosibirsk State Technical University, Novosibirsk, Russia

**Keywords:** dataset, DTI, fMRI, multimodal MRI, olfactory groove meningioma

## Abstract

Olfactory groove meningiomas are uncommon skull base tumors that often present at advanced stages and may cause persistent cognitive and behavioral disturbances despite successful surgical treatment. Neuroimaging studies of this tumor entity have been limited, and publicly available multimodal MRI datasets remain scarce. Here, we present a prospective, single-center dataset comprising multimodal magnetic resonance imaging and longitudinal clinical data from patients with olfactory groove meningiomas acquired before and after surgical intervention. The dataset includes high-resolution structural MRI, diffusion MRI with tensor-derived metrics, resting-state functional MRI, tumor and peritumoral edema segmentation masks, and detailed clinical and neuropsychological assessments. Imaging data were acquired using a standardized protocol and processed with reproducible pipelines, including quality control and de-identification procedures, and organized in a BIDS format. This dataset is intended to support reproducible research and secondary analyses focused on tumor-related brain alterations, imaging biomarker development, and postoperative recovery in neuro-oncology.

## Background and summary

Olfactory groove meningiomas (OGMs) are an uncommon subgroup of skull base tumors, typically benign and slow-growing, accounting for approximately 6%–18% of all intracranial meningiomas (1). Due to their indolent growth pattern, OGMs often remain clinically silent for extended periods, resulting in diagnosis at advanced stages when tumors have already reached substantial size (2). Patients commonly present with headaches, visual disturbances, cognitive impairment, and a wide spectrum of neuropsychiatric symptoms, including affective and behavioral changes (3). Previous studies have reported persistent cognitive and behavioral deficits in a subset of patients even after complete surgical resection, suggesting that tumor-related brain alterations may not be fully reversible (4).

Magnetic resonance imaging (MRI) is central to the diagnosis and management of OGMs, providing detailed anatomical characterization of the anterior cranial fossa and adjacent frontal lobe structures (2). Tumor size, peritumoral edema, and mass effect on surrounding brain tissue are generally considered key determinants of clinical symptomatology (3). Prior neuroimaging studies have primarily focused on tumor morphometry, demonstrating associations between larger tumor volumes and increased severity of neuropsychiatric manifestations (5, 6). Peritumoral edema has also been implicated as an important contributor to cognitive and behavioral disturbances (7). Recent clinical studies have provided further insight into surgical strategies and outcomes in patients with OGMs, contributing to a more comprehensive clinical understanding of this entity (8–10). However, the broader impact of OGMs on brain organization and microstructural integrity remains insufficiently explored.

Advanced neuroimaging techniques, including diffusion and functional MRI, offer the opportunity to investigate alterations in brain tissue and network organization beyond visible structural abnormalities (11). In parallel, artificial intelligence has demonstrated promising performance in brain tumor imaging analysis, particularly in tasks such as automated segmentation, tumor characterization, and prediction of clinical outcomes (12, 13). While resting-state functional connectivity has been extensively studied in psychiatric and neurological disorders, systematic neuroimaging investigations of brain functional and microstructural changes in patients with OGMs remain scarce. There is a lack of publicly available, multimodal neuroimaging datasets combining high-resolution structural MRI, diffusion MRI, and longitudinal clinical assessments in this patient population.

To address this gap, we present a prospective, single-center dataset of patients with olfactory groove meningiomas acquired before and after surgical intervention. The dataset includes high-resolution structural MRI, diffusion MRI with calculated tensor-derived metrics, resting-state fMRI, tumor and peritumoral edema segmentation masks, and detailed clinical and neuropsychological assessments. Imaging data were acquired using a standardized protocol and processed using reproducible, openly available pipelines, with careful quality control and de-identification procedures. Longitudinal follow-up data allow investigation of postoperative changes and recovery trajectories.

This dataset is intended as a resource for the neuroimaging and neuro-oncology communities to facilitate studies of tumor-related brain alterations, methodological benchmarking, and the development of imaging biomarkers related to tumor burden, peritumoral edema, and surgical outcome. By providing well-characterized multimodal MRI data in a relatively understudied tumor entity, this work aims to support reproducible research and enable secondary analyses across a broad range of clinical and computational applications.

## Methods

### Participants and study design

This prospective study was carried out at our hospital from January 2023 to February 2026 and aimed at examining neuroimaging and neuropsychological findings associated with olfactory groove meningioma, as well as identifying predictors of surgical outcomes. The study included a total of 37 participants after all the exclusion criteria were applied (see Supplementary Figure S1 for the study flow chart). All patients were diagnosed with olfactory groove meningioma and referred for surgical intervention (ages 37–73, 78% female).

The study included patients with OGMs, who had no MRI contraindications, no intracranial or facial anomalies, and no history of neurological or psychiatric disorders. The study excluded participants who had multiple meningiomas. On the first day of their hospital stay, patients underwent MRI examination in accordance with a developed protocol. Additionally, clinical and neuropsychological assessment were conducted on the same day.

The follow-up data of patients were collected after the surgical intervention for 23 patients (at the time of analysis), following the same examination protocol as for presurgical assessment (median follow-up time = 301 days, IQR = 158).

All patients provided written informed consent to participate in the study, in accordance with the Declaration of Helsinki. In addition to obtaining consent for participation, all participants provided explicit consent for data sharing as part of this study. Ethics approval for the research protocol was given by the local ethics committee of the Federal Center of Neurosurgery, dated 27-09-2021 (protocol # 9).

### MRI acquisition

MR imaging data were acquired via a 3T system (Ingenia, Philips Healthcare, The Netherlands) equipped with a 16-channel receiver head coil. Imaging was conducted according to a multiparametric high-resolution brain MRI protocol, including the following:

1. High-resolution 3D T1-weighted gradient echo imaging with the following acquisition parameters: TR, 6.57 msec; TE, 2.95 msec; and flip angle, 8 degrees. Images were obtained in the sagittal plane with a nominal voxel size of 0.5 × 0.5 × 0.5 mm^3^ after interpolation (matrix 256 × 256 × 340, field-of-view 256 × 256 × 170 mm), with no partial Fourier transform. The actual voxel size was 1 × 1 × 1 mm^3^. The protocol included 2 runs for each subject (before and after contrast injection), and the scanning time was 5 min per run.
2. High-resolution 3D T2-weighted turbo spin echo imaging with the following acquisition parameters: TR, 3500 msec; TE, 300 msec; and flip angle, 90 degrees. Images were obtained in the sagittal plane with a nominal voxel size of 0.5 × 0.5 × 0.5 mm^3^ after interpolation (matrix 256 × 256 × 360, field-of-view 256 × 256 × 180 mm), with no partial Fourier transform. The actual voxel size was 1 × 1 × 1 mm^3^. The scanning time was 5 min 18 s
3. Diffusion tensor imaging (DTI) with the following acquisition parameters: TR - 12 s, TE - 110 msec, 64 diffusion directions with b = 1500 and one volume with b = 0, phase encoded direction anterior to posterior. Images were obtained in the axial plane with an actual voxel size of 2 × 2 × 2 mm^3^ without interpolation (matrix 128 × 126 × 44, field-of-view 256 × 256 × 144 mm, covering the entire brain), with partial Fourier transform. Additionally, three short sequences were obtained with b = 0 only (one with a phase-encoded direction anterior to posterior and two with a phase-encoded direction posterior to anterior) because they are necessary for subsequent spatial distortion correction. The total scanning time was 12 min.
4. Resting-state functional MRI (rs-fMRI) was conducted with the following parameters: EPI-BOLD sequence, TR – 3 s, TE – 30 msec, flip angle – 90 degrees, 200 volumes (plus 10 dummy scans), and eyes closed. Images were obtained in the axial plane with an actual voxel size of 3 × 3 × 3 mm^3^ without interpolation (matrix 80 × 80, field-of-view 240 × 240 × 135 mm, covering the entire brain), with partial Fourier transform. The scanning time was 10 min.

**Figure 1:**
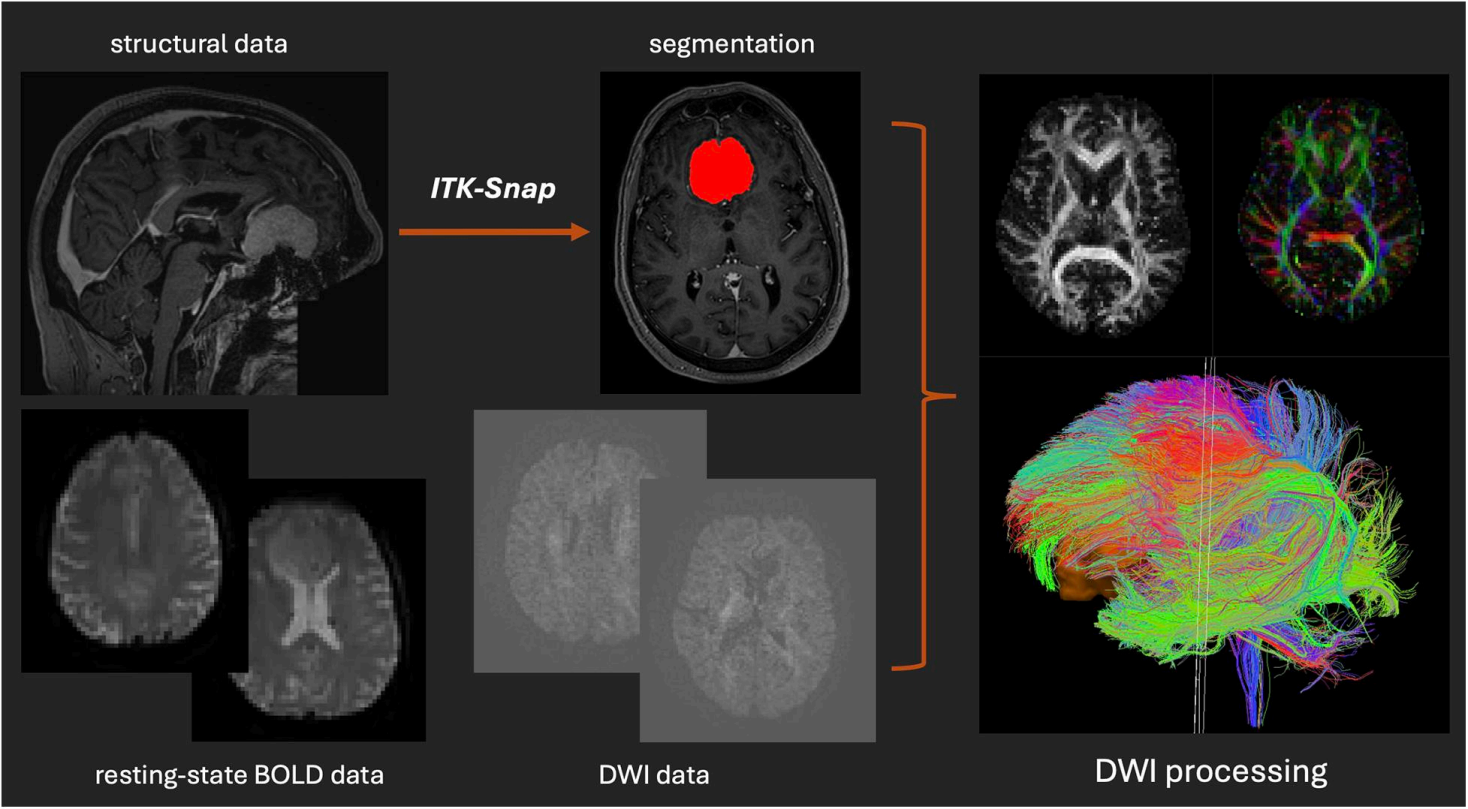
Overview of the multimodal MRI dataset and data processing workflow, including raw structural, DWI, and resting-state fMRI data, T1w–based tumor segmentation, and diffusion modeling with tensor metrics and constrained spherical deconvolution tractography.

An example of the project's MRI data is presented in Figure 1. Additionally, conventional sequences, including FLAIR, DWI, and SWI (not presented here), were obtained to exclude other brain pathologies.

### MRI preprocessing

All raw MRI scans were first evaluated by an experienced neuroradiologist to identify tumor and ensure concordance between imaging findings and the diagnosis. In the second step, structural images, including T1-weighted (pre- and post-contrast) and T2-weighted series, were visually inspected to exclude scans with prominent artifacts. Only cases with acceptable diagnostic quality proceeded to the next workflow stage.

In the second step, the whole dataset was reorganized into a BIDS (https://bids.neuroimaging.io/) with dcm2bids software (https://github.com/UNFmontreal/Dcm2Bids). Structural series (T1w and T2w) were defaced with Pydeface software (https://github.com/poldracklab/pydeface). The acquired structural MR images were subsequently subjected to a quality assessment via the MRI-QC package - https://mriqc.readthedocs.io/en/latest/.

### Tumor and peritumoral edema segmentation

For each patient, the olfactory groove meningioma was segmented with ITK-Snap software (version 4.0.0, http://www.itksnap.org) via a semiautomatic classification algorithm. Tumor segmentation was performed on postcontrast T1-weighted images, referred to as T2-weighted images. After that, perilesional edema segmentation was performed (when presented) based on T2-WI. The segmentation results were saved as binary masks in NifTI format with subsequent volume calculation.

### DTI data processing

Diffusion MRI data preprocessing and analysis were performed using a combination of tools from the FMRIB Software Library (FSL, version 6.0.6) and ExploreDTI (version 4.8.6). The processing pipeline was designed to correct for susceptibility-induced distortions, eddy-current effects, and subject motion, followed by tensor-based and higher-order diffusion modeling.

#### Distortion and motion correction

Susceptibility-induced geometric distortions were corrected using FSL's *topup* tool ((https://web.mit.edu/fsl_v5.0.10/fsl/doc/wiki/topup.html), exploiting pairs of non-diffusion-weighted (b = 0) images acquired with opposite phase-encoding directions (anterior–posterior and posterior–anterior). Following susceptibility correction, eddy-current–induced distortions and subject motion were corrected using FSL's *eddy* tool (https://fsl.fmrib.ox.ac.uk/fsl/docs/diffusion/eddy/index.html). The eddy correction included outlier detection and replacement to mitigate signal dropouts caused by motion or physiological artifacts. Gradient directions were rotated accordingly to preserve diffusion encoding consistency.

#### Tensor fitting and diffusion metrics extraction

Diffusion tensor estimation was performed in ExploreDTI software (https://www.exploredti.com) using a linear least-squares fitting approach. Tensor fitting was conducted voxel-wise on the corrected diffusion data, with appropriate handling of diffusion gradient orientation information. From the fitted diffusion tensors, standard scalar diffusion metrics were calculated, including fractional anisotropy (FA), mean diffusivity (MD), radial diffusivity (RD), and axial diffusivity (AD). All diffusion metric maps were generated in the native diffusion space and stored in NIfTI format for subsequent analyses.

#### Constrained spherical deconvolution processing

Constrained spherical deconvolution (CSD) was performed using ExploreDTI software (https://www.exploredti.com). Fiber orientation distributions (FODs) were estimated using a single-fiber response function derived from voxels with high anisotropy. CSD reconstruction was carried out using a harmonic order appropriate for the acquisition scheme (64 diffusion directions, b = 1500 s/mm^2^). The resulting FODs were visually inspected to ensure biologically plausible fiber orientation patterns, particularly in regions affected by tumor mass effect and peritumoral edema. All diffusion-derived maps and CSD outputs were retained to allow downstream analyses.

### Clinical assessment and surgical interventions

All patients underwent a comprehensive preoperative clinical evaluation. The collected baseline data included presenting symptoms, documented comorbid conditions, body mass index, and the estimated duration of disease prior to intervention. Histopathological examination of the resected specimens was performed to determine the tumor's morphological type and WHO grade.

Functional status was quantified using the Karnofsky Performance Scale (ranging from 0 to 100), assessed both preoperatively and postsurgery. A standardized ophthalmological examination, including visual acuity assessment, was conducted for every patient. Visual acuity is presented in the dataset as a single value between 0 and 1 (per patient), representing the best corrected acuity from either eye, where 0 signifies complete blindness and 1 signifies normal vision.

A detailed neuropsychological battery was administered at three distinct time points: 1) preoperatively, 2) on postoperative days 5–6, and 3) at a long-term follow-up no earlier than six months after surgery. The assessment encompassed the Montreal Cognitive Assessment (MoCA) for general cognition, the Positive and Negative Syndrome Scale (PANSS), the Hospital Anxiety and Depression Scale (HADS), the Montgomery–Åsberg Depression Rating Scale, and the Apathy Evaluation Scale. Specific cognitive domains were evaluated using the WAIS subtests for forward and backward digit span (working memory) and letter as well as category verbal fluency tasks (executive function).

All patients included in the dataset underwent surgical resection via a lateral supraorbital approach. Key surgical and perioperative parameters were recorded, including the duration of the surgical procedure, estimated intraoperative blood loss, and the extent of resection according to Simpson's grading scale. Surgical complications were systematically documented. Postoperative course was characterized by the total length of hospital stay and the patient's neurological status at the time of discharge.

### Data records

The dataset is freely available on the open access OpenNeuro platform in BIDS format and includes all the required components (https://openneuro.org/datasets/ds007345). Clinical and demographic information for each participant, along with tumor and peritumoral edema volumes, main clinical information (include MoCA score, vision acuity, and Karnofsky performance scale), Simpson grade, and histopathological data are provided in the **“participants.tsv”** file. The variables are detailed in the **“participants.json”** file.

The entire dataset includes structural and functional MRI volumes for 37 patients with OGMs, organized in folders labeled sub-***. Furthermore, 6-month follow-up MRI data are available for 22 patients stored in folders labeled sub-***fu.

Each participant's directory has the following folders:

- the **“anat”** folder contains high-resolution defaced structural images (T1w before and after contrast injection, and T2w);
- the **“func”** folder includes functional resting-state BOLD-EPI MRI data (4D data with 200 volumes per participant, excluding dummy scans);
- the **“dwi”** folder contains diffusion tensor imaging data with 64 diffusion directions (*_run-01.nii.gz file) and three b0-only runs with both anterior to posterior (*_run-04.nii.gz) and posterior to anterior (*_run-02.nii.gz and *_run-03.nii.gz) phase-encoded directions relative to the main sequence, acquired for spatial distortion correction with the FSL *topup* procedure (https://web.mit.edu/fsl_v5.0.10/fsl/doc/wiki/topup.html) or alternative software.

All derived data are stored under the derivatives/ directory:

- derivatives/sub-***/segmentation includes the *tumor and peritumoral edema segmentation masks*.
- derivatives/sub-***/dwi_processing contains preprocessed DWI data, including volumes corrected for susceptibility and eddy-current distortions (topup + eddy), fractional anisotropy (FA), radial diffusivity (RD), longitudinal diffusivity (LD), and mean diffusivity (MD) maps, as well as tractography files derived from constrained spherical deconvolution (CSD).
- derivatives/mriqc contains the complete output generated by MRIQC (https://github.com/nipreps/mriqc), including image quality metrics, summary reports, and visual quality assessment files for the structural and functional MRI data.
- derivatives/clinical_data.csv file includes additional surgical (Simpson grade, surgical complications, surgery duration, blood loss, and hospital stay) as well as clinical and neuropsychological information (KPS and MoCA scores before and after surgery, HADS and PANSS scores, clinical symptoms, and olfactory status), which are available for all included participants.

**Figure 2:**
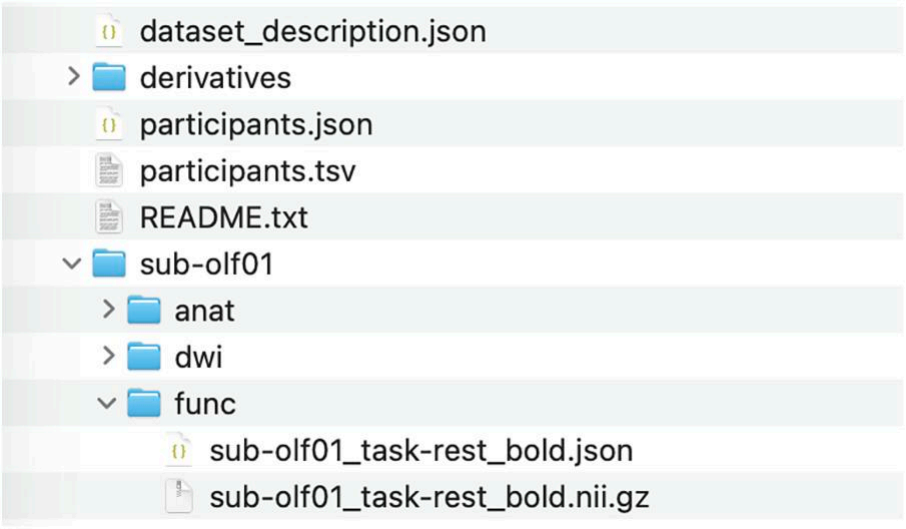
Structure of the dataset shown for one participant.

Figure 2 shows the data structure. Further details about our dataset can be found in the **“README.txt”** file.

## Technical validation

### MRI data quality assessment

The MR images were analyzed via MRIQC software (https://github.com/nipreps/mriqc) to obtain quantitative image quality metrics. For structural images, metrics including the coefficient of joint variation (CJV), contrast-to-noise ratio (CNR), signal-to-noise ratio (SNR), and intensity non-uniformity (INU) were calculated. For functional images, metrics included entropy focus criterion (EFC), foreground–background energy ratio (FBER), full width at half maximum (FWHM), SNR, and temporal SNR (tSNR). For diffusion-weighted imaging, quality assessment included motion-related parameters, SNR of b0 images, EFC, and FBER.

Summary statistics (mean ± SD) of the main MRIQC-derived metrics for each modality are provided in Supplementary Tables S1–S4 (T1-weighted, T2-weighted, diffusion-weighted, and resting-state fMRI, respectively). Representative group-level visualizations are shown in Supplementary Figures S3–S6.

The MRIQC reports for the fMRI data were visually inspected to identify any noticeable artifacts, including aliasing, distortion, and ghosting. The outputs of MRIQC for the entire dataset are available in the **“derivatives/mriqc”** directory in both .tsv and .html formats.

### Sensitivity power analysis

Given the rarity of olfactory groove meningiomas and the absence of prior multimodal MRI datasets providing effect size estimates for *a priori* power calculation, a *post hoc* sensitivity power analysis was conducted. The analysis was performed using the pwr package in R, version 4.5.2. Parameters were set as follows: two-tailed tests, *α* = 0.05, and target power = 0.80. Minimal detectable effect sizes were calculated separately for three analytic designs: (1) paired t-test for longitudinal preoperative-to-postoperative comparisons within the same patient (*n* = 37 pairs); (2) unpaired two-sample t-test for cross-sectional comparisons between patients and healthy controls (*n* = 37 per group); and (3) Pearson correlation analysis within the patient group (*n* = 37). A suitable control dataset is available from a previously conducted study with healthy participants acquired using the same MRI protocol and comparable age distribution (14), enabling future case-control analyses. The results of the sensitivity power analysis are presented below and in Supplementary Figure S2.

For paired comparisons (preoperative vs. postoperative within the same patient, *n* = 37 pairs), the study is powered to detect a moderate effect of Cohen's d ≥ 0.47. For unpaired two-sample comparisons (patients vs. healthy controls, *n* = 37 per group, total *N* = 74), the minimal detectable effect is Cohen's d ≥ 0.66, corresponding to a moderate-to-large effect. For correlation analyses within the patient group (*n* = 37), the minimal detectable Pearson's r is 0.44 (moderate correlation).

These detectable effect sizes are consistent with or exceed those reported in previous neuroimaging and surgical studies of olfactory groove meningiomas (typical *n* = 15–25 per group). The sample size is therefore adequate for the descriptive and exploratory analytic goals of this data report, including longitudinal, case-control, and correlational analyses. Smaller effects may remain undetected, and null findings should be interpreted cautiously.

## Code availability

We utilized openly available code to run FSL's *topup* and *eddy*, as well as MRIQC without generating any specific scripts for the data analysis.

## Data Availability

The datasets presented in this study can be found in online repositories. The names of the repository/repositories and accession number(s) can be found below: https://openneuro.org/datasets/ds007345.

## References

[1] Kasper EM Mirza FA Kaya S Walker R Starnoni D Daniel RT Surgical morbidity in relation to the surgical approach for olfactory groove meningiomas—a pooled analysis of 1016 patients and proposal of a new reporting system. Brain Sci. (2023) 13(6):896. 10.3390/BRAINSCI13060896 37371375 PMC10296289

[2] Damante MA Magill ST Kreatsoulas D McGahan BG Finger G Hatef J A modern approach to olfactory groove meningiomas. J Neurosurg. (2023) 140(5):1215–22. 10.3171/2023.8.JNS2318 37948686

[3] Pashkov A Filimonova E Poptsova A Martirosyan A Prozorova P Moysak G Cognitive, affective and behavioral functioning in patients with olfactory groove meningiomas: a systematic review. Neurosurg Rev. (2025) 48(1):1–16. 10.1007/S10143-025-03626-7 40439793

[4] Lévêque S Derrey S Martinaud O Gérardin E Langlois O Fréger P Superior interhemispheric approach for midline meningioma from the anterior cranial base. Neurochirurgie. (2011) 57(3):105–13. 10.1016/j.neuchi.2011.08.001 21907362

[5] Barzaghi LR Spina A Gagliardi F Boari N Mortini P. Transfrontal-sinus-subcranial approach to olfactory groove meningiomas: surgical results and clinical and functional outcome in a consecutive series of 21 patients. World Neurosurg. (2017) 101:315–24. 10.1016/j.wneu.2017.02.039 28213192

[6] Nanda A Maiti TK Bir SC Konar SK Guthikonda B. Olfactory groove meningiomas: comparison of extent of frontal lobe changes after lateral and bifrontal approaches. World Neurosurg. (2016) 94:211–21. 10.1016/J.WNEU.2016.06.101 27373938

[7] Bassiouni H Asgari S Stolke D. Olfactory groove meningiomas: functional outcome in a series treated microsurgically. Acta Neurochir. (2007) 149(2):109–21. 10.1007/S00701-006-1075-Z 17180303

[8] Bander ED Pandey A Yan J Giantini-Larsen AM Schwartz A Estin J Olfactory groove meningiomas: supraorbital keyhole versus orbitofrontal, frontotemporal, or bifrontal approaches. J Neurosurg. (2023) 140(6):1568–75. 10.3171/2023.10.JNS231432 38064694

[9] Brown NJ Pennington Z Patel S Kuo C Chakravarti S Bui NE Surgical approaches to resection of olfactory groove meningiomas: comparative meta-analysis of the endoscopic endonasal versus transcranial and unilateral versus bilateral approaches. J Neurol Surg B Skull Base. (2024) 86(2):208. 10.1055/A-2297-9055 40104542 PMC11913544

[10] Yilmaz H Akcay E Tabanli A Bologur O Ak C Benek HB Is unilateral extended pterional craniotomy adequate instead of bicoronal (bifrontal) craniotomy in large or giant olfactory groove meningiomas? Turk Neurosurg. (2025) 35(1):56–61. 10.5137/1019-5149.JTN.46246-24.3 39651883

[11] Filimonova E Pashkov A Poptsova A Moysak G Martirosyan A Prozorova P Reorganization of brain networks in olfactory groove meningioma patients: a pilot resting-state fMRI study. Front Neurol. (2025) 16:1644138. 10.3389/FNEUR.2025.1644138/BIBTEX 40948647 PMC12425792

[12] Ozenbas C Engin D Altinok T Akcay E Aktas U Tabanli A. ChatGPT-4o's performance in brain tumor diagnosis and MRI findings: a comparative analysis with radiologists. Acad Radiol. (2025) 32(6):3608–17. 10.1016/j.acra.2025.01.033 39924377

[13] Xu N Ma W Dong W Yin B. A scoping review of artificial intelligence applications in meningioma from image analysis to prognostic prediction. Discover Oncology. (2026) 17(1):319. 10.1007/S12672-026-04476-5 41577884 PMC12909632

[14] Filimonova E Pashkov A Tiutiunnik A Moysak G Martirosyan A Kurilov V A large-scale dataset of pre- and postsurgical MRI data from patients with chronic trigeminal neuralgia. Sci Data. (2025) 12(1):2024. 10.1038/s41597-025-06311-y 41387477 PMC12749398

