# Supplementary material for: Multimodal brain MRI and clinical data in olfactory groove meningioma: a prospective data report
Source: Front Radiol. 2026 May 8;6:1809871. doi: 10.3389/fradi.2026.1809871 (PMC13194104; doi:10.3389/fradi.2026.1809871)
Supplement: Supplementary file 1 [file Datasheet1.pdf]

Supplementary data

Figure S1. Study flow diagram illustrating patient inclusion and exclusion. A total of 54 patients with olfactory groove meningioma were enrolled between January 2023 and February 2026. Seventeen participants were excluded due to incomplete data (related to patients' severe clinical condition), multiple meningiomas, prior surgery, or significant comorbidities. The final cohort comprised 37 patients with multimodal MRI and neuropsychological data before surgical intervention, including follow-up multimodal MRI scans for 23 patients. Two patients were excluded from follow-up due to late post-surgical complications, five declined multimodal follow-up MRI, and seven are scheduled for follow-up scanning.

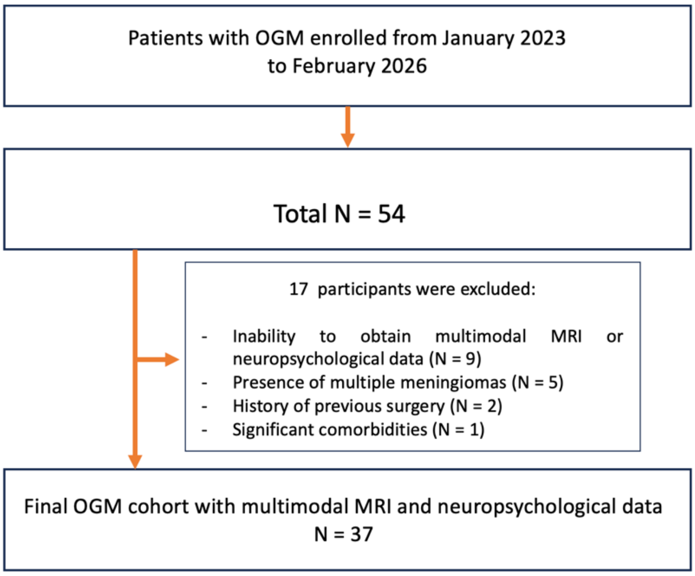

Figure S2. Sensitivity power analysis. (A) Paired t-test for longitudinal comparisons (preoperative vs postoperative, n = 37 pairs). (B) Unpaired t-test for cross-sectional comparisons (patients, n = 37 vs healthy controls, n = 37). (C) Pearson correlation within the patient group (n = 37). Dashed horizontal lines indicate the target power level of 0.80. Dotted vertical lines indicate the minimal detectable effect sizes for each analysis. Shaded curves represent power as a function of the underlying effect size or correlation.

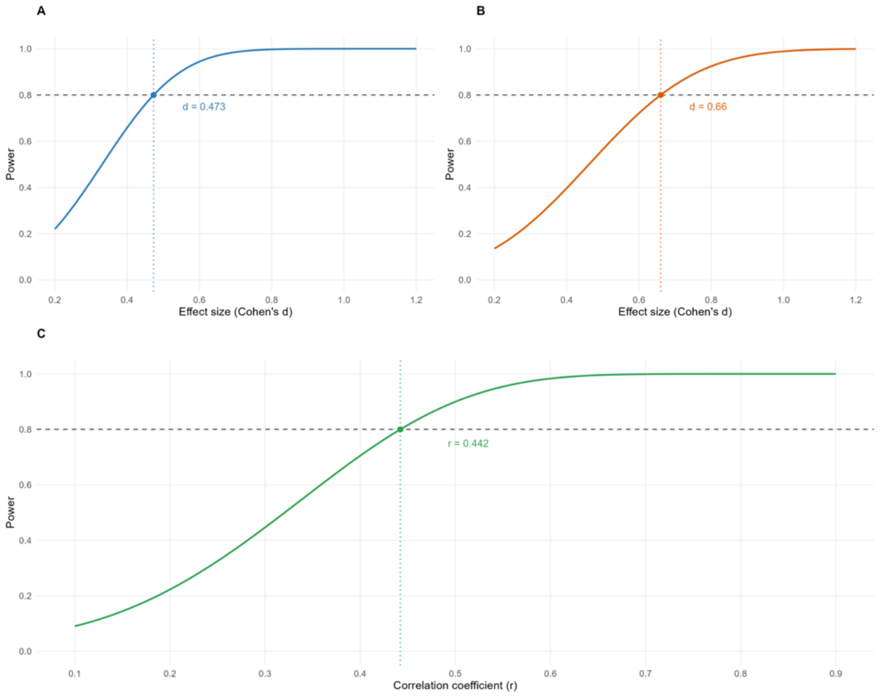

Summary of MRIQC main quality metrics (Mean ± SD) across all modalities

1. T1-weighted images quality metrics

Metric	Mean ± SD	Description
SNR (CSF)	1.40 ± 0.32	Signal-to-noise ratio in cerebrospinal fluid
SNR (GM)	3.70 ± 0.84	Signal-to-noise ratio in gray matter
SNR (WM)	6.93 ± 1.67	Signal-to-noise ratio in white matter
CNR	1.19 ± 0.45	Contrast-to-noise ratio between gray and white matter
EFC	0.73 ± 0.02	Entropy focus criterion
FBER	3244.89 ± 5684.67	Foreground–background energy ratio
INU (median)	0.80 ± 0.08	Median intensity non-uniformity
INU (range)	0.40 ± 0.11	Range of intensity non-uniformity

Table S1. MRIQC-derived quality metrics for 3D T1-weighted images. SNR (signal-to-noise ratio) is reported separately for cerebrospinal fluid (CSF), gray matter (GM), and white matter (WM). CNR (contrast-to-noise ratio) reflects tissue contrast between gray and white matter. EFC (entropy focus criterion) is sensitive to motion-related artifacts and image blurring. FBER (foreground–background energy ratio) reflects signal strength relative to background noise. INU (intensity non-uniformity) metrics describe the magnitude and spatial variability of bias field effects. Overall, the metrics indicate stable image quality with adequate signal and contrast for downstream analysis.

2. T2-weighted images quality metrics

Metric	Mean ± SD	Description
SNR (CSF)	1.63 ± 0.30	Signal-to-noise ratio in cerebrospinal fluid
SNR (GM)	4.08 ± 1.18	Signal-to-noise ratio in gray matter
SNR (WM)	5.16 ± 1.68	Signal-to-noise ratio in white matter
CNR	0.53 ± 0.26	Contrast-to-noise ratio

EFC	0.60 ± 0.02	Entropy focus criterion; reflects motion and blurring
FBER	245.30 ± 210.53	Foreground–background energy ratio
INU (median)	0.94 ± 0.03	Median intensity non-uniformity
INU (range)	0.44 ± 0.09	Range of intensity non-uniformity

Table S2. MRIQC-derived quality metrics for T2-weighted images. SNR (signal-to-noise ratio) is reported for cerebrospinal fluid (CSF), gray matter (GM), and white matter (WM). CNR (contrast-to-noise ratio) reflects tissue contrast and is typically lower in T2-weighted imaging. EFC (entropy focus criterion) captures motion-related artifacts. FBER (foreground–background energy ratio) reflects signal strength relative to background noise. INU (intensity non-uniformity) metrics describe bias field magnitude and variability. Overall, the metrics indicate consistent image quality across subjects.

3. DTI quality metrics

Metric	Mean ± SD	Description
Framewise displacement (FD)	27.65 ± 18.57	Motion-related displacement between volumes (scale-dependent, used comparatively)
SNR (b0)	5.75 ± 1.29	Signal-to-noise ratio of non-diffusion-weighted images
EFC	0.32 ± 0.02	Entropy focus criterion
FBER	$6.99 \times 10^6 \pm 3.77 \times 10^7$	Foreground–background energy ratio

Table S3. MRIQC-derived quality metrics for diffusion-weighted imaging (DWI). FD (framewise displacement) reflects relative subject motion between volumes (due to varying image contrast across diffusion directions and model-based correction, FD values in DWI should be used for relative quality assessment rather than absolute motion quantification). SNR (b0) represents baseline signal quality of non-diffusion-weighted images. EFC (entropy focus criterion) captures motion and image degradation. FBER (foreground–background energy ratio) reflects signal relative to background noise and may show high variability in diffusion imaging. Overall, the metrics indicate acceptable data quality, with variability reflecting susceptibility effects and tumor-related anatomical distortion.

4. fMRI quality metrics (resting-state BOLD)

Metric	Mean ± SD	Description
Framewise displacement (FD)	0.43 ± 0.29	Subject motion during scanning (mm)
DVARS	1.16 ± 0.09	Temporal signal variability across volumes
SNR	3.07 ± 0.27	Signal-to-noise ratio of BOLD signal
tSNR	39.42 ± 12.15	Temporal signal-to-noise ratio
EFC	0.40 ± 0.02	Entropy focus criterion; reflects motion and blurring
FBER	158914.71 ± 67169.87	Foreground–background energy ratio

Table S4. MRIQC-derived quality metrics for resting-state functional MRI. FD (framewise displacement) reflects subject motion (in mm), while DVARS quantifies temporal signal variability. SNR and tSNR (temporal signal-to-noise ratio) describe signal quality and time-series stability. EFC (entropy focus criterion) captures motion-related artifacts, and FBER (foreground–background energy ratio) reflects signal strength relative to noise. The observed values indicate stable and reliable functional MRI data suitable for downstream analysis.

MRIQC group level boxplots (Figures S3-S6) show the median (central line), interquartile range (box), whiskers extending to 1.5 × interquartile range, and individual subject values (dots)

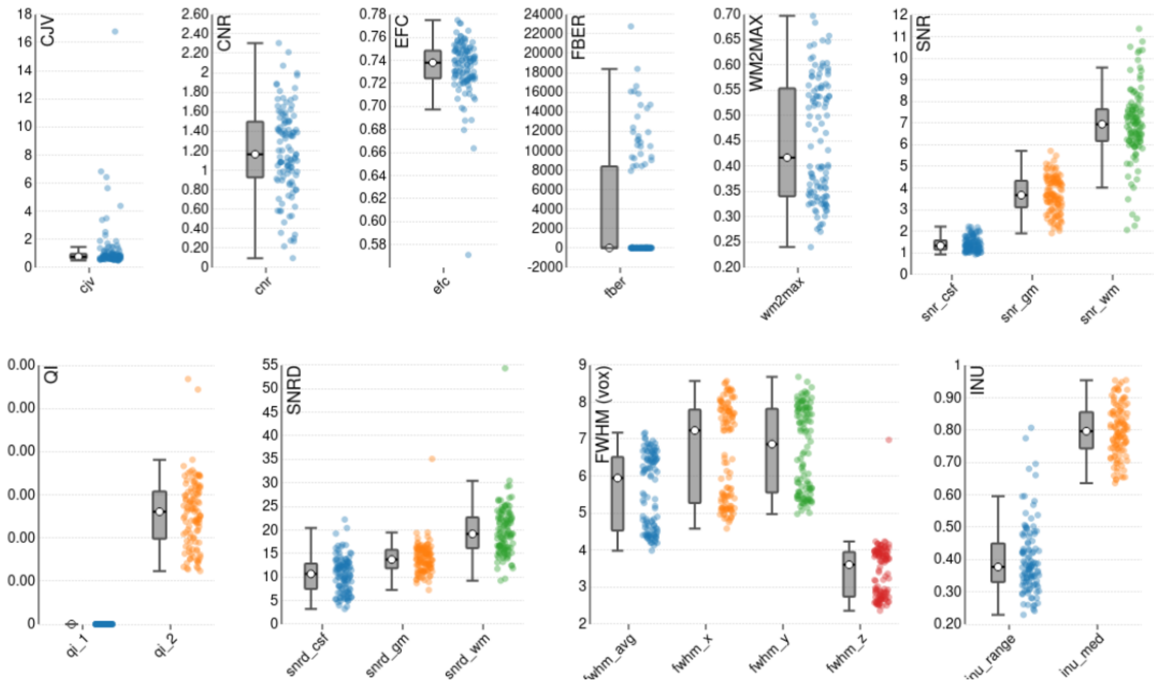

Figure S3. The results of MRIQC group analysis for T1-weighted structural MRI data. CJV - coefficient of joint variation; CNR - contrast-to-noise ratio; EFC - entropy focus criterion; FBER - foreground-background energy ratio; WM2MAX - white-matter to maximum intensity ratio; SNR - signal-to-noise ratio; QI - quality index, including fraction of non-brain voxels classified as artifacts (q1) and residual structured noise in background (q2); SNRD - Dietrich's SNR values for CSF (snrd_csf), gray matter (snrd_gm), white matter (snrd_wm); FWHM - estimated image smoothness (full width at half maximum), including the average

value (fwhm_avg), and directional estimates along the x-, y-, and z-axes (fwhm_x/y/z); INU - intensity non-uniformity in the image, including the range (inu_range) and median (inu_med) across the brain

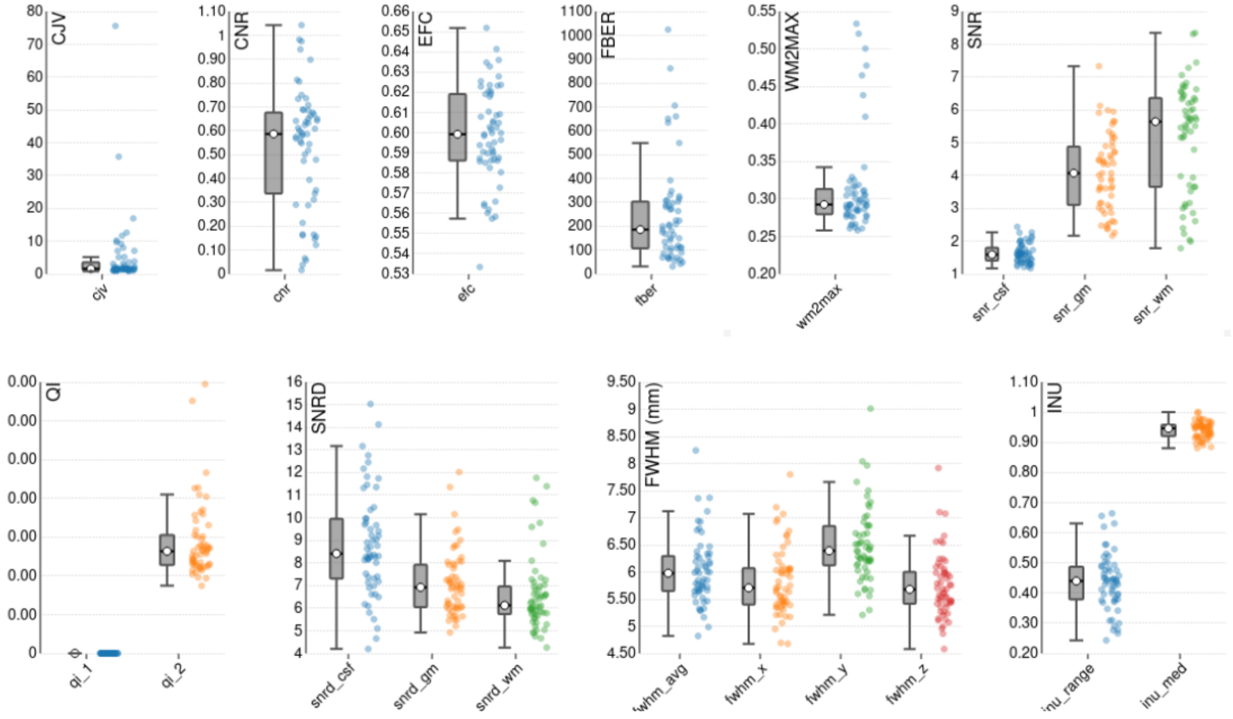

Figure S4. The results of MRIQC group analysis for T2-weighted structural MRI data. CJV - coefficient of joint variation; CNR - contrast-to-noise ratio; EFC - entropy focus criterion; FBER - foreground-background energy ratio; WM2MAX - white-matter to maximum intensity ratio; SNR - signal-to-noise ratio; QI - quality index, including fraction of non-brain voxels classified as artifacts (q1) and residual structured noise in background (q2); SNRD - Dietrich's SNR values for CSF (snrd_csf), gray matter (snrd_gm), white matter (snrd_wm); FWHM - estimated image smoothness (full width at half maximum), including the average value (fwhm_avg), and directional estimates along the x-, y-, and z-axes (fwhm_x/y/z); INU - intensity non-uniformity in the image, including the range (inu_range) and median (inu_med) across the brain.

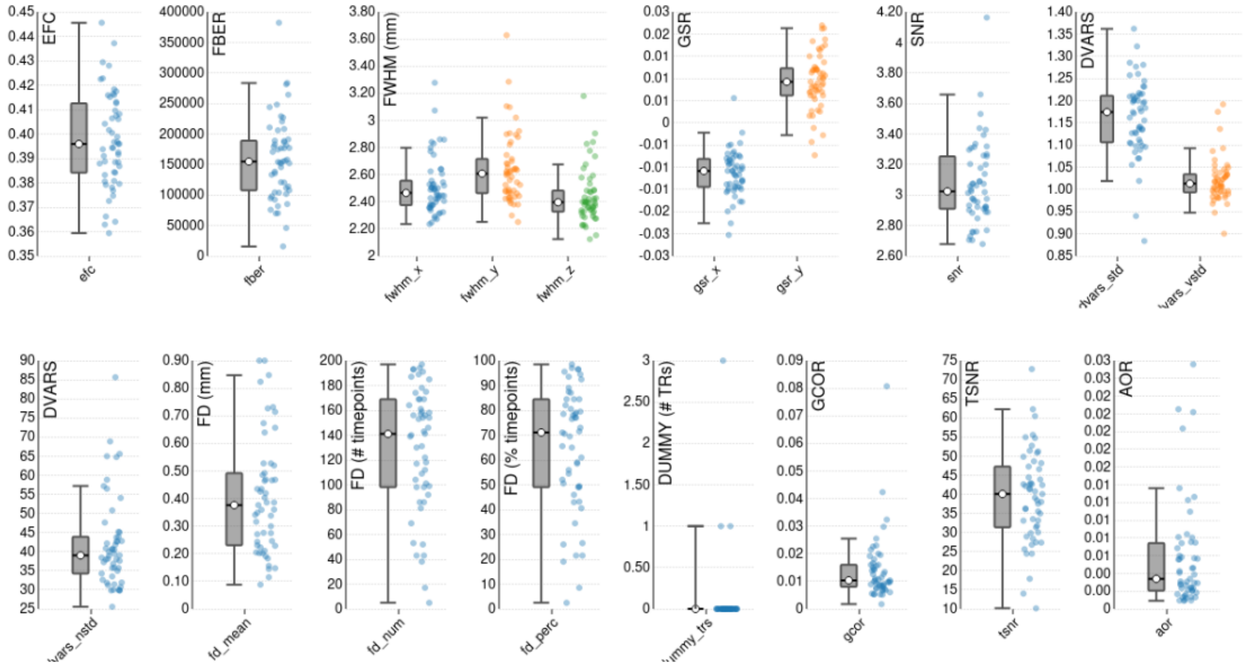

Figure S5. The results of MRIQC group analysis for functional MRI data. EFC - entropy focus criterion; FBER - foreground-background energy ratio; FWHM - estimated image smoothness for the x-, y-, and z-directions (fwhm_x/y/z); GSR - global signal regression estimates along the x and y axes (gsr_x,y); SNR - signal-to-noise ratio; DVARS - temporal derivative of RMS variance over voxels, including standard (dvars_std), variance-normalized (dvars_vstd), and non-normalized (dvars_nstd) measures; FD - framewise displacement, including absolute displacement in millimeters - FD (mm), number of timepoints above threshold - FD (# timepoints), and percentage of timepoints above threshold - FD (% timepoints); DUMMY - number of initial dummy TRs discarded; GCOR - global correlation across all brain voxels; TSNR - temporal signal-to-noise ratio; AOR - AFNI’s outlier ratio.

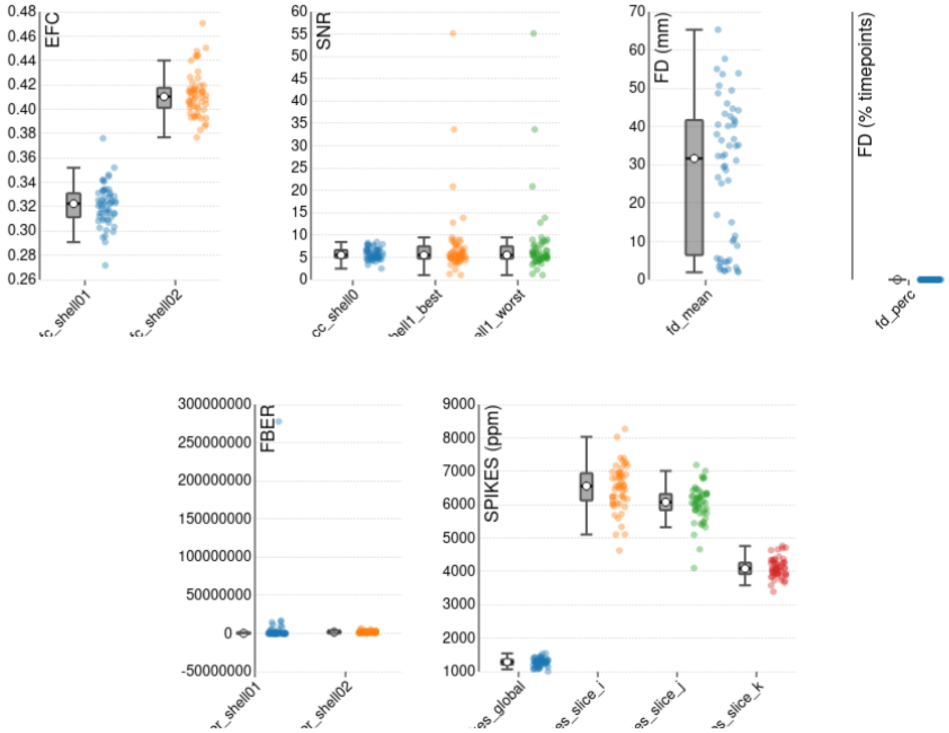

Figure S6. The results of MRIQC group analysis for diffusion-weighted MRI data. EFC - entropy focus criterion per shell, including shell01 and shell02 (efc_shell01,02); SNR - signal-to-noise ratio per shell, including shell0 (snr_shell0), best (snr_best), and worst (snr_worst) volumes; FD - framewise displacement in millimeters - FD (mm) and percentage of timepoints above threshold - FD (% timepoints); FBER - foreground-to-background energy ratio per shell, including shell01 and shell02 (fber_shell01,02); SPIKES - fractions of voxels classified as spikes, including global (spikes_global) and slice specific counts along i-, j-, and k-axes (spikes_slice_i,j,k).
